# HIV-1 Integrase-DNA Recognition Mechanisms

**DOI:** 10.3390/v1030713

**Published:** 2009-11-05

**Authors:** Jacques J. Kessl, Christopher J. McKee, Jocelyn O. Eidahl, Nikolozi Shkriabai, Ari Katz, Mamuka Kvaratskhelia

**Affiliations:** Center for Retrovirus Research and Comprehensive Cancer Center, College of Pharmacy, The Ohio State University, Columbus, OH 43210, USA; E-Mails: kessl.1@osu.edu (J.J.K.); mckee.473@osu.edu (C.J.M.); eidahl.1@osu.edu (J.O.E.), shkriabai.1@osu.edu (N.S.); katz.147@osu.edu (A.K.)

**Keywords:** HIV, retroviruses, DNA, integrase, crosslinking, footprinting

## Abstract

Integration of a reverse transcribed DNA copy of the HIV viral genome into the host chromosome is essential for virus replication. This process is catalyzed by the virally encoded protein integrase. The catalytic activities, which involve DNA cutting and joining steps, have been recapitulated *in vitro* using recombinant integrase and synthetic DNA substrates. Biochemical and biophysical studies of these model reactions have been pivotal in advancing our understanding of mechanistic details for how IN interacts with viral and target DNAs, and are the focus of the present review.

## 1. Introduction

HIV-1 integrase (IN) catalyses integration of the reverse transcribed DNA copy of the viral genome into a host chromosome (reviewed in [1]), a step which is essential for the retroviral lifecycle. Integrase selectively recognizes and synapses the two viral DNA ends to form a catalytically competent nucleoprotein complex. Understanding of structural and mechanistic foundations for IN-viral DNA interactions have been the subject of intense research as both a fascinating biological paradigm and an important therapeutic target for the development of antiretroviral therapies. Practical benefits of these efforts have been manifested by the recent discovery of the strand transfer inhibitors (STI) and a successful launch of Raltegravir into the clinic. Strikingly, STIs selectively bind the preformed IN-viral DNA complex rather than free protein [2–4], thus exemplifying the significance of detailed characterization of the functional nucleoprotein complexes.

## 2. DNA Processing and Joining Reactions Catalyzed by HIV-1 IN

The integration of viral DNA into a host chromosome involves two chemical reactions. In the first step, which is called 3′-processing and takes place in the cytoplasm shortly after the viral DNA is made, IN hydrolyzes a GT dinucleotide from each 3′ end of the viral DNA. In the second step, IN catalyzes concerted integration of the processed viral DNA ends into chromosomal DNA. The sites of attack on the two target DNA strands are separated by 5 bp for HIV-1 IN, which leads to DNA strand dissociation in the small double-stranded DNA fragment between the attachment sites. The subsequent repair of the intermediate species by cellular enzymes completes the integration reaction.

**Figure 1.**
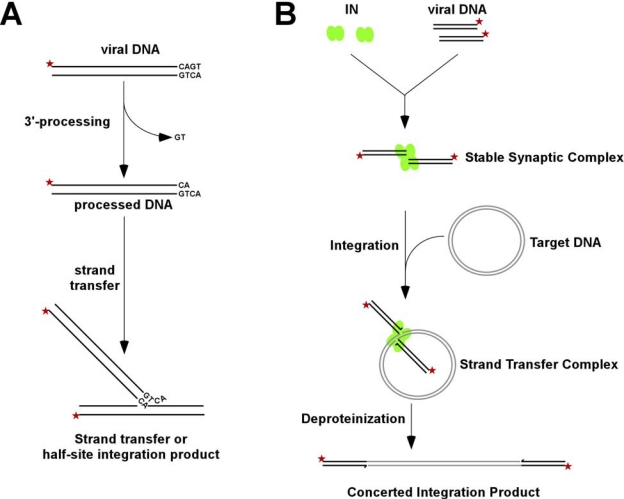
Schematic presentation of HIV-1 IN activity assays *in vitro*. **(A)** These reactions are typically performed with purified recombinant IN and 21-mer double-stranded DNA mimicking the U5 sequence. The enzyme first removes the GT dinucleotide from the 3′-terminal, and then covalently joins the recessed 3′-end to the target DNA. In these reactions the U5 sequence serves as both viral and target DNA. The strand transfer products result from integration of only one viral DNA end into the target DNA, while pair-wise integration products are not observed. **(B)** The concerted integration assays and critical nucleoprotein intermediates. Selective interaction of IN with viral DNA ends results in a highly stable nucleoprotein complex termed the stable synaptic complex (SSC). Next, IN in the context of SSC engages with the target DNA to form the strand transfer complex (STC), which carries out the concerted integration reaction. These nucleoprotein complexes are readily separated by native agarose gel electrophoresis. Deproteinization of the STC leads to the formation of the concerted integration product. The asterisks in A and B indicate the P^32^ labeled 5′-end of viral DNA.

In infected cells IN functions in the context of a large nucleoprotein complex termed the preintegration complex (PIC), where a number of viral and cellular proteins contribute to retroviral integration [5–17]. PICs can be extracted from infected cells and used for biochemical assays *in vitro* [18–25]. However, the amounts of these nucleoprotein complexes are not sufficient to perform atomic structural or even lower resolution biophysical analyses. Therefore, recombinant IN and model DNA substrates have been employed instead to study protein-nucleic acid interactions. Typically, purified recombinant protein and short DNA substrates (∼21-mer dsDNA mimicking the U5 end of viral DNA) are utilized to monitor 3′-processing and strand transfer activities (Figure 1A). These reactions, however, do not yield concerted integration products and instead, result in integration of one viral DNA end into the target DNA. More recently, assays using a longer donor DNA substrate of several hundred basepairs and a second circular target DNA have been devised, which allow effective concerted integration of two viral DNA ends [26–31] (see Figure 1B). This improved experimental design has furthermore allowed isolation and characterization of critical nucleoprotein intermediates that are reminiscent of IN-viral DNA interactions in the PIC in the infected cells [27,28].

Along with the biologically relevant 3′-processing and strand transfer activities, purified IN exhibits additional activities *in vitro*. The enzyme can reverse the strand transfer reaction by site selectively cleaving the integrated DNA. This reaction is called disintegration [32]. A recent report has indicated that the recombinant protein can also catalyze internal cleavage at a palindromic sequence mimicking LTR-LTR junction [33,34]. However, there is no evidence as yet that these additional catalytic activities observed *in vitro* can also occur in infected cells.

## 3. Structure of HIV-1 Integrase

**Figure 2.**
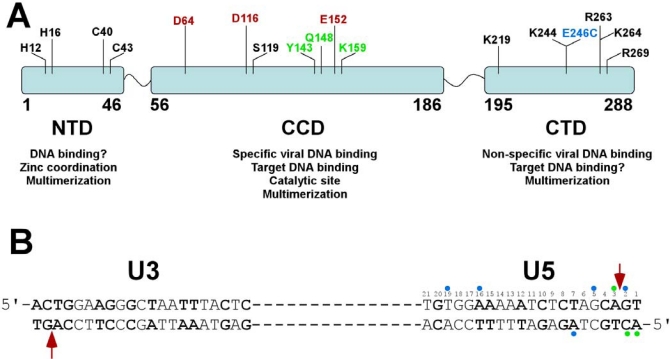
**(A)** Schematic presentation of the three domain structure of HIV-1 IN. The NTD residues (H12, H14, C40 and C43) coordinate Zn and contribute to the functional multimerization. It is not entirely clear whether the NTD directly binds viral or target DNA. The CCD contains the catalytic DDE motif. This domain interacts with both viral and target DNA. A number of residues (Y143, Q148 and K159) selectively interact with terminal U5 bases, while S119 has been implicated in direct interactions with the target DNA. The CCD is also critical for the functional multimerization. The CTD is highly basic and non-specifically interacts with viral DNA. Several CTD residues implicated in viral DNA binding are indicated. It remains to be determined whether the CTD could also coordinate the target DNA. **(B)** Sequences of U3 and U5 termini of viral DNA. The base-pairs that are identical in U3 and U5 sequences are in bold. A majority of IN-viral DNA mapping experiments used the U5 sequence and the interacting sites are indicated with circles. Note color coordination between the residues in A and respective nucleotide positions in B. The catalytic residues in A and the arrow pointing to the specific cleavage sites at U3 and U5 termini are in red. The CCD amino acids Y143, Q148 and K159 (colored green) have been shown to selectively crosslink with the terminal nucleotides marked with green circles [60]. The E246C mutant is colored blue and its multiple crosslinking sites [61] in viral DNA are depicted by blue circles.

IN consists of three distinct structural and functional domains: the N-terminal domain (NTD), the catalytic core domain (CCD) and the C-terminal domain (CTD) (Figure 2A). Each domain directly or indirectly contributes to IN-DNA interactions. The NTD, which encompasses residues 1–46, is linked to the CCD (residues 56–186) through a linker segment of aa 47–55. Another flexible loop comprised of residues 187–194 connects the CCD with the CTD (residues 195–288). Results of structural biology studies revealed each individual domain as a dimer [35–39]. More recent two-domain crystal structures comprised of the CCD and CTD [40] or NTD and CCD [41] likewise unveiled dimeric organizations. However, dimer interfaces for individual NTDs [35] and CTDs [38] differ from those observed in the two domain fragments [40,41] and it is not clear how these two domains are assembled in full-length oligomeric IN. In contrast, the CCD-CCD interactions have remained consistent in various constructs [36,37,40–42] suggesting that these protein-protein contacts are likely to be also preserved in the fully functional nucleoprotein complex. Structural analyses of the full-length recombinant IN or its complexes with model DNA substrates have not been amenable to crystallographic and NMR approaches.

The NTD has a HTH fold that is conserved in all retroviral and retrotansposon integrases [35,41]. It contains conserved pairs of histidine (H12, H16) and cysteine (C40 and C43) residues that bind zinc [35,43] and contributes to functional oligomerization of IN [44,45]. The mutations of Zn binding residues yield monomeric IN and inhibit the 3′-processing and strand transfer reactions [46,47]. Furthermore, recent biochemical and structural studies implicated the NTD-CCD interactions in functional tetramerization of IN [47,48]. The K14A substitution at the putative NTD-CCD interface destabilized IN tetramers and compromised IN catalytic activities [48].

The CCD belongs to a superfamily of polynucleotidyl transferases that share an overall fold of bacterial RNase H and exhibit a similar catalytic mechanism [36]. HIV-1 IN catalytic site is comprised of the invariant triad of acidic residues D64, D116 and E152 that act by binding divalent metal ions [49,50]. Mutations of these residues severely compromise IN activities *in vitro* and in infected cells [46,51,52]. Biochemical assays with purified IN revealed that it requires either Mg^2+^ or Mn^2+^ to carry out the reactions with model DNA substrates. Of these, Mg^2+^ is considered to be the physiological cofactor due to its relative abundance in the cells. Several structural studies have shown a single divalent metal bound to the active site of the HIV-1 CCD [37]. However, based on the two-metal mechanism for structurally and functionally similar polynucleotidyl transferases [53,54], it has been proposed that DNA binding stabilizes the second metal in the active site [55]. IN uses the same catalytic site for 3′-processing and strand transfer reactions. Therefore, the CCD is likely to harbor both viral and target DNA binding sites. Furthermore, the CCD is also an essential building block for formation of the functional multimeric IN. The CCD-CCD interface is fairly large (∼1,650 Å^2^) and mutations destabilizing these interactions adversely affect IN catalytic activities [36,41].

The C-terminal domain (CTD) is rich in basic amino acids and adopts an SH3-like fold [38]. Other proteins with the same fold bind the minor groove of DNA in a nonspecific manner [56–58]. Similarly, the CTD is thought to provide a stabilizing platform for DNA substrates. In addition, the CTD has been implicated in functional oligomerization of IN. L241A and L242A mutations along the C-terminal dimer disrupted IN dimerization and compromised catalytic activities [59].

## 4. Sequence and Structure of Viral DNA

IN productively binds U5 and U3 termini of viral DNA (Figure 2B). Footprinting of PICs isolated from the infected cells revealed the terminal 200–250 base pairs of each viral DNA end as primary protein binding sites [19]. In contrast, internal regions of the viral DNA did not exhibit strong protein binding. DNase I digestion of the stable synaptic complex assembled with purified IN and long DNA substrates implicated much smaller segments of viral DNA. Only terminal 16 and 32 bps were protected in the SSCs assembled with the W235H mutant and wild type IN, respectively [27,62]. The significantly larger footprint observed in the nucleoprotein complexes isolated from infected cells could probably be explained by contributions of other viral and cellular proteins associated with the PICs [19].

Biochemical studies have shown that recombinant IN exhibits comparable affinities with respect to specific and non-specific DNA sequences. Oligodeoxynucleotides with random sequences can effectively compete with IN-viral DNA interactions and impair the 3′-processing reactions [63–65]. In the context of infected cells this inherent property of IN is unlikely to significantly deter the retroviral protein from its biological target, the viral DNA ends, as the assembly of the PICs takes place in the cytoplasm where competition from non-specific DNA sites are likely to be minimal. Once bound to the viral DNA, however, IN forms a very stable nucleoprotein complex [28]. Divalent metal has been shown to contribute to assembly and stabilization of HIV-1 IN-viral DNA complex [66–71].

Functional assays have shown that IN can distinguish between the viral DNA ends and nonspecific substrates. Mutational studies *in vitro* and *ex vivo* have indicated the importance of CA/TG dinucleotide pair for effective 3′-processing of the viral DNA ends [63]. Additional proximal regions of viral DNA have also been implicated in specific recognition of the viral DNA [60]. Mutations at positions 11–13 from the U5 terminus substantially compromised 3′-processing activities of recombinant IN in the presence of Mg^2+^ ions with lesser affects being observed with Mn^2+^, suggesting a differential effect of divalent metals on sequence specific binding. Another study [72] identified positions 17–20 to be important for effective concerted integration *in vitro*. At the same time these experiments indicated that HIV-1 IN could tolerate significant divergence in the viral DNA sequences.

One important feature contributing to selective recognition of the LTR termini by IN could be the DNA end distortion. NMR analysis of a 17 base pair oligonucleotide containing the U5 terminal sequence revealed that base stacking and minor groove were significantly disordered at the cleavage site [73]. The chemical footprinting of the avian sarcoma virus (ASV) IN complex with cognate DNA, moreover, revealed that protein binding further destabilized the terminal three base pairs [74]. Significantly, the authors observed a good correlation between DNA end distortion and cleavage activities [74]. Introducing mismatch bases at the terminal three positions enhanced base unstacking and unpairing, and substantially stimulated the site specific processing activities.

The alternative experimental strategies to identify the LTR regions important for selective recognition involved application of DNA analogs. Probing effects of various DNA backbone, base, and groove modifications on IN catalytic activities suggested that IN requires flexibility of the phosphodiester backbone at the scissile bond [75]. The other study examined 2′-modified nucleosides and 1,3-propanediol insertions in various positions of the U5 sequence [76]. Akin to the mutagenesis experiments [60] divalent metal dependent effects were observed upon altering certain regions of the DNA [76]. Nucleoside modifications at positions 3, 5 and 6 significantly diminished Mg^2+^ dependent activities, while Mn^2+^ dependent reactions were less affected. In contrast, Mg^2+^ and Mn^2+^ dependent activities were equally impaired when the modifications were introduced at positions 7–9 [76]. Taken together, the biochemical approaches enabled the delineation of several important features of viral DNA essential for formation of the functional nucleoprotein complexes. Nevertheless, the detailed mechanism for selective recognition remains elusive. Ideally, atomic structures of IN complexes with specific and non-specific DNAs would be necessary to fully address this question.

## 5. Mapping HIV-1 IN-Viral DNA Interactions

IN functions as a multimer. Mutagenesis experiments have shown that two inactive mutants of IN with substitutions in different domains of the protein can be combined to regain the catalytic function [77–79]. These results have indicated that different monomers within the IN multimer provide complementary rather than symmetrical contacts to DNA [77–79].

At the sub- to low-micromolar concentrations of IN normally used in the *in vitro* activity assays, the protein exists as a mixture of monomers, dimers and tetramers in the absence of DNA [64,80–82]. Interactions between individual subunits are highly dynamic in the unliganded IN [48], but are stabilized by DNA binding [27]. Time resolved fluorescence anisotropy measurements indicated that individual IN subunits bind viral DNA in a cooperative manner with a stoichiomery of two IN monomers bound to each viral DNA end [34,83]. Small angle X-ray scattering experiments have also indicated that monomeric IN could assemble onto a short specific DNA as dimers and effectively catalyze 3′-processing reactions [84]. These studies have suggested that a dimeric IN could suffice to process one viral DNA end [85,86].

A number of studies have suggested that a tetramer of IN synapses the two viral DNA ends into the fully functional nucleoprotein complex. Crosslinking experiments have revealed IN tetramers as a dominant species in the nuclear extracts of infected cells [14]. Consistently, the stable synaptic complexes assembled *in vitro* contained a tetrameric form of IN [27]. Efforts to more directly visualize the size of the nucleoprotein complexes included atomic force microscopic analysis of ASV IN in its free form and in the complex with cognate short DNA, which also demonstrated substrate-induced assembly of the IN tetramer [87]. Similar results were obtained by electron microscopy and singe-particle image reconstruction of HIV-1 IN complex with a model DNA junction mimicking the pairwise integration structure [88]. Other studies [62,89], however, proposed that a higher order oligomer (for example, octamer) of IN could be formed during the concerted integration. We will return to discussion of IN oligomeric states later in the context of IN interactions with its principal cellular cofactor lens epithelium derived growth factor (LEDGF/p75).

To identify IN amino acids directly interacting with DNA substrates photo and chemical cross-linking studies have been conducted [60,89–93]. These experiments revealed several key contact points. For example, the CCD residues (K159, Q148 and Y143) have been shown to specifically tether with the nucleotide analogs incorporated at the terminal portion of the viral DNA ends [60,90]. K159 is part of the helix containing the catalytic E152 and could directly interact with viral DNA. Y143 and Q148 are situated in the flexible loop and could contribute to accurate positioning of viral and target DNA substrates. Consistent with this, Pommier and co-workers have found that the STI 1-(5-chloroindol-3-yl)-3-hydroxy-3-(2*H*-tetrazol-5-yl)-propenone effectively interrupted the disulfide cross-linking between Q148C and the C2 of viral DNA, suggesting the importance of these nucleoprotein contacts for the strand transfer step [92].

IN-viral DNA crosslinking experiments have also implicated a number of the CTD residues in interactions with distal segments of the LTR [60,89,93,94]. The reactive bases introduced in the region centered at 6–7 base-pairs from the U5 terminal were found to effectively tether with the CTD amino acids. For example, Gao *et al.* observed a strong crosslink between the E246C mutant and position A7 [94]. However, analysis of additional DNA positions (G2, G5, A7, G16 and G19) conducted by our group revealed comparable reactivity of E246C with all the substrates examined [61]. The latter results are consistent with the non-specific mode of the CTD-DNA interactions and indicate that the exact locations of the CTD in the functional nucleoprotein complex could not be reliably determined from these experiments. Indeed, even though the U5 sequence has been used in these experiments, IN could bind with equal affinity to specific and non-specific ends of the 21-mer double stranded DNA thus differently positioning the CTD on the DNA. It should also be noted that majority of the CTD contacts implicated in DNA binding are lysines and arginines [61], which could potentially engage in charge-charge interactions with the phosphate backbone of viral DNA.

Sequence alignments between HIV and other retroviral INs have also been exploited for identification of IN amino acids contributing to viral DNA recognition. Leis and coworkers introduced several ASV IN residues at analogous positions in HIV-1 IN and monitored whether these substitutions altered their preferences for LTR sequences [95,96]. HIV-1 IN residues that changed specificity included V72, S153, K160, I161, G163, V165, H171 and L172 suggesting that these amino acids could directly or indirectly contribute to viral DNA recognition. In separate studies highly conserved HIV-1 IN residues were targeted by site directed mutagenesis to evaluate their roles for virus replication [97,98]. The authors grouped the mutations that solely affected the integration step in class I, while the substitutions that exhibited additional assembly and/or reverse transcription defects were placed in class II. Overall, these *ex vivo* experiments [97,98] have been instrumental for dissecting the functionally essential residues and validating the biological importance of a number of amino acids identified from *in vitro* analysis of model IN-viral DNA complexes.

Several lines of evidence have emerged that IN undergoes significant conformational change upon DNA binding. Our mass spectrometry (MS) based footprinting experiments have uncovered DNA induced structural rearrangement involving the flexible loop between the CCD and CTD [61]. Bushman and coworkers have detected differential cross-linking of CTD residues with blunt ended and processed DNA substrates, suggesting protein structural changes upon cleavage of the viral DNA terminus [94]. Asante-Appiah and Skalka have revealed a metal dependent-conformational rearrangements, which affected the recognition of the CCD and CTD, but not the NTD, by domain selective antibodies [99]. Roth and co-workers have found that the functional IN tolerated the insertion of a 19 amino acid sequence at the helix connecting the CCD and CTD [100]. These observations collectively point to the importance of the linker loop of aa 187–194 (Figure 2A) for providing much needed flexibility to the CCD and the CTD to productively assemble onto viral DNA.

## 6. HIV-1 IN Interactions with the Target DNA

In common with other retroviruses HIV-1 IN exhibits a weak primary sequence preference for integration sites [101–107]. While in cells different retroviruses display distinguishable integration site preferences, the target DNA sequence is probably a minor contributor to this. In the case of HIV-1 the interactions of the retroviral enzyme with chromatin are strongly mediated by the cellular transcription coactivator LEDGF/p75 and active genes are favored for integration (see [108, 109] for recent reviews and the following chapter).

*In vitro* experiments have indicated that a wide variety of DNA sequences could serve as targets for the stand transfer reactions [101–104]. At the same time a number of studies noted preferential integration in distorted DNA sites [110,111]. For example, *in vitro* ASV and HIV INs primarily targeted sites adjacent to stem loop structures in a plasmid DNA cruciform [112]. The importance of the target DNA distortion for effective integration has also been noted in the context of chromatinized templates [110–114]. DNA assembled into nucleosomes was more favorable for integration than naked DNA with the most bent regions of DNA on the nucleosomes being preferentially targeted [110–114].

To identify IN residues interacting with the target DNA, Katzman and co-workers used an elegant approach in which they compared sequence variations in patient-derived HIV-1 integrases with alterations in the preferred integration sites in the target DNA and identified a small number of amino acids substitutions [115]. These substitutions were then examined for their interactions with the target DNA *in vitro*. These experiments have clearly delineated the importance of HIV-1 IN residue S119 for the target site selection, while the substitutions at this position did not affect IN interactions with viral DNA [115]. More recent efforts from the same group have extended the target DNA binding platform to include 5 additional CCD amino acids [116].

Earlier crosslinking studies have suggested that the NTD and the CCD could also interact with the target DNA [89,93]. However, these experiments were performed with the dumbbell DNA, which is a substrate for disintegration rather than for 3′-processing or stand transfer reaction. Furthermore, detailed mutagenesis studies of the NTD and the CTD residues [46,48,98,117–119] failed to identify phenotypes resembling to those observed with S119 substitutions [115]. Mutations of functionally significant residues in the NTD and CTD equally impaired 3′-processing (which does not involve interactions with the target DNA) and stand transfer activities. Therefore, it remains obscure whether the NTD and the CCD could directly contribute to target DNA binding.

## 7. Concerted Integration Intermediates

The majority of biochemical and biophysical studies reviewed above have been conducted using recombinant IN and short DNA substrates and revealed important details for IN-DNA interactions. However, these reaction conditions yield integration of only one viral DNA end into the target DNA (termed as half-site integration), rather than concerted integration of a pair of viral DNA ends (termed as full-site integration) as occurs *in vivo*. More recently, modifications of reaction conditions allowed effective full-site integration of two viral DNA ends [26–31]. The most notable change in the assay has been the replacement of short DNAs with a longer donor DNA substrate (∼1 kbps) and a second circular target DNA (compare Figures 1 A and B). It is unclear why longer donor DNA substrates are favored for the pair-wise integration, given that IN selectively binds only a small terminal region of viral DNA. It has also been noted that preprocessed DNAs preferentially yield half-site reaction products, while the blunt-ended DNA substrates are more efficient for full-site integration [26].

The optimized reaction conditions allowed Li *et al.* to isolate and characterize critical nucleoprotein intermediates involved in the pair-wise integration [27,28]. Using the azido-containing aryl β-diketo acid inhibitor the authors effectively trapped the first important reaction intermediate, the stable synaptic complex formed between a tetramer of IN and two viral DNA ends. Particularly noteworthy is the observation that the SSC is as stable as the integrase complex with viral DNA assembled in the context of the PIC [27,28]. The SSC effectively resists treatments with the buffers containing high ionic strength or detergents. In contrast, stable nucleoprotein complexes are not formed upon IN interactions with a single viral DNA end in reactions that lead to half-site integration or with long DNAs that lack the LTR sequence [27,28].

The 3′-processing reactions take place within the SSC. IN remains stably associated with the pair of viral DNA ends and engages target DNA to form a second stable complex termed the strand transfer complex (STC) [27,28]. This complex carries out concerted integration of the pair of viral DNA ends into target DNA. Li *et al.* have monitored the reaction time course with two-dimensional gel electrophoresis and found that two DNA strand transfer steps occur sequentially and exhibit slow kinetics [27]. At early reaction time points the authors detected the intermediate species, which contained one viral DNA covalently integrated into the target DNA, while the other viral DNA was non-covalently held within the STC. At the later time points, however, essentially all the STC contained both viral DNA ends integrated, suggesting that once the correct nucleoprotein complex is assembled, the concerted integration is highly efficient [27].

## 8. LEDGF/p75 Strongly Modulates HIV-1 IN-DNA Interactions

LEDGF/p75 is a principal binding partner for HIV-1 and other lentiviral INs and markedly enhances the integration process in the infected cells (see [109] for recent review). The cellular protein functions as a bifunctional tether: its C-terminal part contains integrase binding domain (IBD) that directly engages lentiviral IN, while the N-terminal part tethers the PICs to the chromatin. *In vitro* experiments carried out with purified proteins and model DNA substrates indicated that LEDGF/p75 strongly modulates strand transfer activities [30,47,48,120,121]. In the reactions with short donor DNA substrates and circular target plasmid, LEDGF/p75 potently enhanced both half-site and concerted integration reactions [47,120,122]. However, in the assays with long donor DNA substrates, the cellular cofactor almost exclusively stimulated integration of only one viral DNA end [30,48]. The reasons for different outcomes with short and long donor DNA substrates are not understood.

Interestingly, order-of-addition experiments performed with long donor DNA indicated that sub-stoichiometric amounts of LEDGF/p75 added to the preformed IN-viral DNA complex modestly stimulated concerted integration [30]. However, preincubation of LEDGF/p75 with IN and subsequent addition of viral DNA to the reaction selectively impaired concerted integration, whereas the half-site strand transfer was markedly elevated [30]. While the structural basis for these observations has been obscure, initial clues have emerged from recent MS footprinting and x-ray crystallographic studies of IN-LEDGF/p75 interactions [47,48].

**Figure 3.**
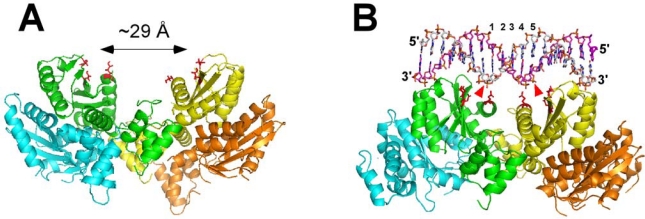
Molecular models for two distinct forms of HIV-1 IN tetramers. **(A)** An “open” conformation of tetrameric IN. This model is based on the HIV-1 IN two domain (NTD-CCD) structure [41] and our MS footprinting results [48] indicating that such a conformation is stabilized by LEDGF/p75 in the absence of viral DNA. This “open” conformation of tetrameric IN could catalyze 3′-processing and half-site integration reactions, however an incorrect spacing (∼29 Å) between the two active sites would hamper the concerted integration. **(B)** A “closed” conformation of HIV-1 IN tetramer. This model was built by Hare *et al.* using one of the crystal forms of the MVV IN structure [47], where the catalytic sites are positioned optimally for concerted integration. It has been proposed that this structure could be stabilized by two viral DNAs [47]. However, viral DNAs have not been included in the model. Instead, the relative positioning of two catalytic sites with respect to the target DNA is shown to demonstrate the 5 bps separation consistent with a pair-wise integration. Red arrows point to the target scissile bonds. Individual subunits are colored cyan, green, yellow and orange. Side chains of catalytic residues in green and yellow subunits are depicted in red. For clarity only NTD-CCD fragments are depicted, while the CTDs, which are also present in these models [47,48], are not shown.

Our group has shown that direct binding of LEDGF/p75 or LEDGF IBD strongly stabilizes highly dynamic interactions of IN subunits and promotes IN tetramerization [48]. Furthermore, MS footprinting experiments identified intra- and inter-protein-protein interactions and enabled detailed modeling of the complex (Figure 3A) [48]. The model has suggested that in the preformed IN-LEDGF/p75 complex a pair of active sites of IN are separated about ∼29 Å, which would enable the retroviral enzyme to effectively catalyze 3′-processing and strand transfer reactions. However, the concerted integration would not be efficient as this distance is larger than the ∼15 Å or 5 bps separation expected between two insertion sites in the target DNA (Figure 3A). This model is consistent not only with *in vitro* functional studies [30,48,121] but also with the observations in infected cells [5,123]. For example, overexpression of the IBD effectively impaired HIV-1 replication in target cells [5,123]. Of note, the IBD was significantly more effective at suppressing HIV-1 replication in LEDGF/p75 deficient cells (555-fold) compared with cells containing normal LEDGF/p75 levels (∼30-fold) [5]. A potential competition between the IBD and endogenous LEDGF/p75 cannot fully explain these observations. Instead, *in vitro* functional assays and MS-footprinting experiments suggest that the IBD binding to IN prior to IN-viral DNA complex formation could stabilize a tetrameric form of IN, which is not fully functional [48]. Collectively, these findings suggest that HIV-1 IN tetramers formed in the IN-viral DNA and IN-LEDGF/p75 complex may not be identical and that the productive integration would require the following sequence of events. Highly dynamic HIV-1 IN subunits first assemble onto two viral DNA ends to form the stable synaptic complex, where two catalytic sites position themselves for pair-wise integration. This IN tetramer-viral DNA complex then binds LEDGF/p75, with the cellular protein directing the PICs to the active genes without significantly affecting the prearranged IN-viral DNA conformations.

Studies with other lentiviral INs have also indicated differential modulation of their function and structure by LEDGF/p75. For example, the cellular cofactor almost exclusively stimulated concerted integration catalyzed by equine infectious anemia virus IN, while the addition of LEDGF/p75 to bovine immunodeficiency virus IN equally enhanced half- and full-site integration products [124]. Recently a co-crystal structure of maedi-visna virus (MVV) IN in the complex with the IBD has been reported, which has revealed four distinct tetrameric forms of this lentiviral IN in the complex with LEDGF/p75 [47]. Of these, in three tetramers the spacing between a pair of DDE motifs was significantly greater (∼ 27 Å) than that required for concerted integration. These findings agree very well with our model for the HIV-1 IN-LEDGF/p75 interactions [48] (also see Figure 3A), and reinforce the notion that relative positioning of the two active sites could indeed be one of the main reasons for differential modulation of strand transfer activities by LEDGF/p75.

Of note, one of MVV IN tetramers observed in the crystallographic studies contained a pair of active sites optimally situated to carry out effective concerted integration reactions [47]. Significant variations in relative positioning and orientations between the two dimers allowed a pair of DDE motifs from opposing CCDs to approach 15 Å separation [47]. The results with MVV IN have been exploited to build a molecular model for the HIV-1 counterpart [47] (also see Figure 3B). Comparison of the two models (Figures 3A and B) reveals “open” and “closed” conformations for IN tetramers. It is now of significant interest to clarify whether there is a correlation between the relative abundance of these two distinct tetrameric forms of IN in the reaction mixture and relative yield of half- and full-site integration products. Studies to test these potential structure-function relationships are currently underway in our group and very likely also in other laboratories.

## 9. Molecular Modeling of the Functional Nucleoprotein Complexes

Crystallographic determination of the two-domain structures prompted molecular modeling research [41,61,89,94,95,125–129]. The two crystal structures [40,41] can be superimposed through the common CCD to generate a plausible model for the full length protein. Biochemical and biophysical results reviewed above have further aided in positioning viral DNA in the multimeric IN. Additional clues for IN-DNA interactions have been provided from the crystal structure of prokaryotic transposase 5 (Tn5) in complex with cognate DNA (reviewed in [130]). Tn5 and HIV-1 IN share the structurally and functionally similar CCDs. Moreover, the crystal structure implicates individual Tn5 subunits in establishing complementary contacts with cognate DNA [130], which parallels well with the asymmetric mode of viral DNA binding to HIV-1 IN protomers [77–79].

A majority of the models generated up to date implicate the IN tetramer in interactions with two viral DNA ends [41,61,94,95,126–129]. Such a stoichiometry for protein-DNA interactions is supported by a number of experimental results [14,27,87,88]. Despite this principal agreement the IN-DNA models obtained by different groups vary significantly in positioning individual protein subunits and domains as well as DNA binding channels in the nucleoprotein complex, indicating that the available experimental data comprises an insufficient number of constraints for formulating a common outcome [41,61,89,94,95,125–129]. Indeed, while there is a good consensus that different monomers provide complementary contacts to viral DNA, it is not clear whether these interactions are enabled by individual subunits within a dimeric IN, or two subunits each from separate dimer contribute to viral DNA binding. Therefore, some modeling studies [61,125,126] employed a strategy where one viral DNA was coordinated to IN dimer and then two IN dimer-viral DNA complexes were assembled together to compose the SSC. Alternative approaches [41,127,128] have considered utilizing IN tetramer as a minimal viral DNA binding platform, where two dimers are stabilized by two viral DNA ends.

The absolute requirement for every modeling analysis has been to position DDE motifs over the respective scissile bond [41,61,89,94,95,125–129]. The crosslinking and mutagenesis data implicating immediate vicinity of the catalytic site in interactions with terminal bases of viral DNA [60,90] are also normally considered in these in silico experiments. Furthermore, the available models implicate the CCD in direct interactions with the target DNA, which is consistent with the experimental findings indicating the role of S119 in the target site selection [115,116].

The published models also agree that the CTD interacts with viral DNA. However, exact positioning of this domain with respect to viral DNA sequence varies significantly. This is not surprising given a non-specific nature of the CTD-DNA interactions observed in crosslinking studies [61]. The available models disagree regarding the role of the CTD in coordinating the target DNA. As discussed above, while earlier crosslinking experiments suggested potential binding of the CTD with the target DNA in the context of the dumbbell DNA, these interactions could not be confirmed by detailed mutational analysis. Therefore, the exact role of the CTD in target DNA binding remains uncertain.

The main inconsistency between different models is in asserting the role of the NTD. In some models the NTD is implicated in direct interactions with viral DNA [61,125], while other studies limit its contributions to protein-protein contacts [41,126–128]. Our MS-based footprinting analysis of the IN-DNA complex has revealed DNA dependent shielding of the surface accessibility of N-terminal K14 [61]. However, protections in the nucleoprotein complex could arise from direct protein-DNA or DNA induced protein-protein interactions. Further site directed analysis from our group [48] clarified the importance of K14 for dimer-dimer interactions, which in turn is essential for formation of the catalytically competent IN tetramer. Our findings [48] have been fully corroborated by more recent crystallographic analysis of the MVV IN-IBD complex [47], which show that the tetrameric structure is stabilized by intermolecular interactions between the NTD of one dimer and the CCD of another dimer. Yet, what configurations the NTDs adopt in the context of the full length protein or its complex with viral and target DNAs remains enigmatic.

The recent two domain structure of MVV integrase tetramer [47], where two active sites are optimally positioned for concerted integration provides a new useful building block for modeling experiments. In fact, Hare *et al.* have been able to superimpose partial HIV-1 integrase structures onto their MVV structure to generate a model of the full-length tetramer devoid of significant steric clashes [47]. The authors have suggested that such a tetramer could be stabilized by the bound DNA, but at the same time, they have acknowledged that the protein could undergo significant conformational change upon viral DNA binding. Thus, the efforts to generate a plausible model for the synaptic complex continue.

## 10. Remaining Questions and Outlook

A wealth of biochemical and biophysical data has been generated over the past two decades and provided insights into HIV-1 IN-DNA recognition mechanisms. Yet, atomic details of the protein-nucleic acid interactions are missing. Instead, the two domain structures of HIV-1 IN have been determined and formed a platform for molecular modeling research. However, a complex nature of the multi subunit arrangements in the functional complex and the asymmetric mode of viral DNA binding have presented a real challenge to generate a consensus model for the IN-viral DNA complex. Where do we go from here? Below we outline a few priority areas as a part of a wider roadmap toward detailed understanding of structural and mechanistic details of HIV-1 integration.

Crystallographic efforts to determine the IN-viral DNA structure are ongoing. Recently, high concentrations of purified IN-DNA complexes were obtained as required for structural determination [131]. For this, Alian *et al.* used soluble mutant IN and disulfide-mediated crosslinking to stabilize the nucleoprotein complex [131]. Significantly, this complex was functionally competent and coordinated STI. Further adjustments may still be required, though, to obtain the complex amenable to atomic analysis. Alternative strategies involve using other retroviral enzymes. For example, recombinant prototype foamy virus IN from the Spumavirus genus is highly soluble and robustly catalyzes the concerted integration reactions with 16-bps substrates [132], thus presenting an intriguing model for detailed structural analysis.

Recent reports have defined a powerful *in vitro* model system for assembly of the SSC that closely mimic IN-viral DNA interactions in PICs [26–31]. A logical continuation of these studies is to scale up the SSC preparations for their subsequent characterization with various biophysical approaches. For example, we are currently analyzing the SSCs with the MS-based footprinting method. Equally, the applications of other experimental tools previously utilized in studies with IN-short DNA complexes can now be extended to probing the concerted integration intermediates. These experiments could shed light on organization of individual protein subunits within the fully functional nucleoprotein complex.

While there is a general consensus that the principal function of LEDGF/p75 is to tether PICs to the chromatin, many important structural and mechanistic details regarding how LEDGF/p75 modulates IN interactions with viral DNA or navigates the SSC through the chromatinized DNA remain to be elucidated. Moreover, we still do not understand why the length of viral DNA so dramatically affects the pair-wise integration. Particularly puzzling are the observations that LEDGF/p75 can stimulate both half- and full-site integrations with short DNA, while the cellular cofactor selectively impairs the concerted integration with long donor DNA substrates. The efforts to further optimize *in vitro* reaction conditions will continue to approach conditions and the efficacy of concerted intergration observed in infected cells. Toward this end a recent study has established *in vitro* conditions, where reconstituted polynucleosomes serve as target acceptor templates for physiologically relevant analysis of the integration process [133]. Further *in vitro* and *ex vivo* experiments are warranted to elucidate important details of how LEDGF/p75 promotes integrase-chromatin interactions.

Recent biochemical and structural studies have indicated the highly flexible nature of IN subunit-subunit interactions, and that assembly of the fully functional nucleoprotein complex requires very accurate interplay between interacting subunits [47,48]. It is intriguing to exploit this complex multi subunit organization as a novel therapeutic target. A broad skepticism for developing small molecule inhibitors for protein-protein interactions can be met with the alternative hypothesis that the potential inhibitors could stabilize inactive conformation of multimeric IN rather than compete with subunit-subunit interactions. A rationale for this is provided by the observations that IBD stabilizes a tetrameric form of IN, which effectively catalyzes 3′-processing and half-site integration, but is selectively impaired for concerted integration [48]. As further proof-of-principal we have recently shown that a small molecule inhibitor can stabilize a functionally compromised multimeric form of HIV-1 IN [134]. Further research in this direction may well lead to the development of new allosteric inhibitors of IN that could complement Raltegravir and other retroviral compounds in treating aids patients.
